# Covid-19 Swept Nursing Homes in the United States

**DOI:** 10.1093/geroni/igaa057.3439

**Published:** 2020-12-16

**Authors:** Lei Yu

**Affiliations:** Miami University, Oxford, Ohio, United States

## Abstract

Nursing Homes experienced a colossal loss impacted by the Covid-19 pandemic in the United States. This study applied Covid-19 Nursing Home Dataset (updated on 08/16/2020) released by Data.CMS.gov to explore possible factors behind the death s at nursing homes. The results indicated 2.55 residents died per week at a nursing home averagely. Besides, the absence of nursing staff, aides, clinical physicians, PPE supplies contributes to more deaths at nursing homes. Lastly, the number of positive COVID-19 cases of nursing home staff positively associate with the number of total deaths of residents（R=0.65). These findings provide more pieces of evidence for nursing home administrators and policymakers to make adjustments to help nursing home residents better cope with challenges caused by the pandemic; however, this dataset is not the final data for the pandmic is not over. Also, the dataset covers few demographic information (gender, race, ethinicty and so on) ; therefore, researchers could explore the relationship between the demographic features and COVID-19 deaths at nursing homes.

